# Supplementary material for: The interactome of intact mitochondria by cross-linking mass spectrometry provides evidence for coexisting respiratory supercomplexes
Source: Mol Cell Proteomics. 2017 Dec 8;17(2):216–32. doi: 10.1074/mcp.RA117.000470 (PMC5795388; doi:10.1074/mcp.RA117.000470)
Supplement: Supplemental Data [file supp_RA117.000470_133922_0_supp_23978_fzffwf.zip › spectra_annotation/mito_spectra_annotation/138-1-9-1-4-1.pdf]

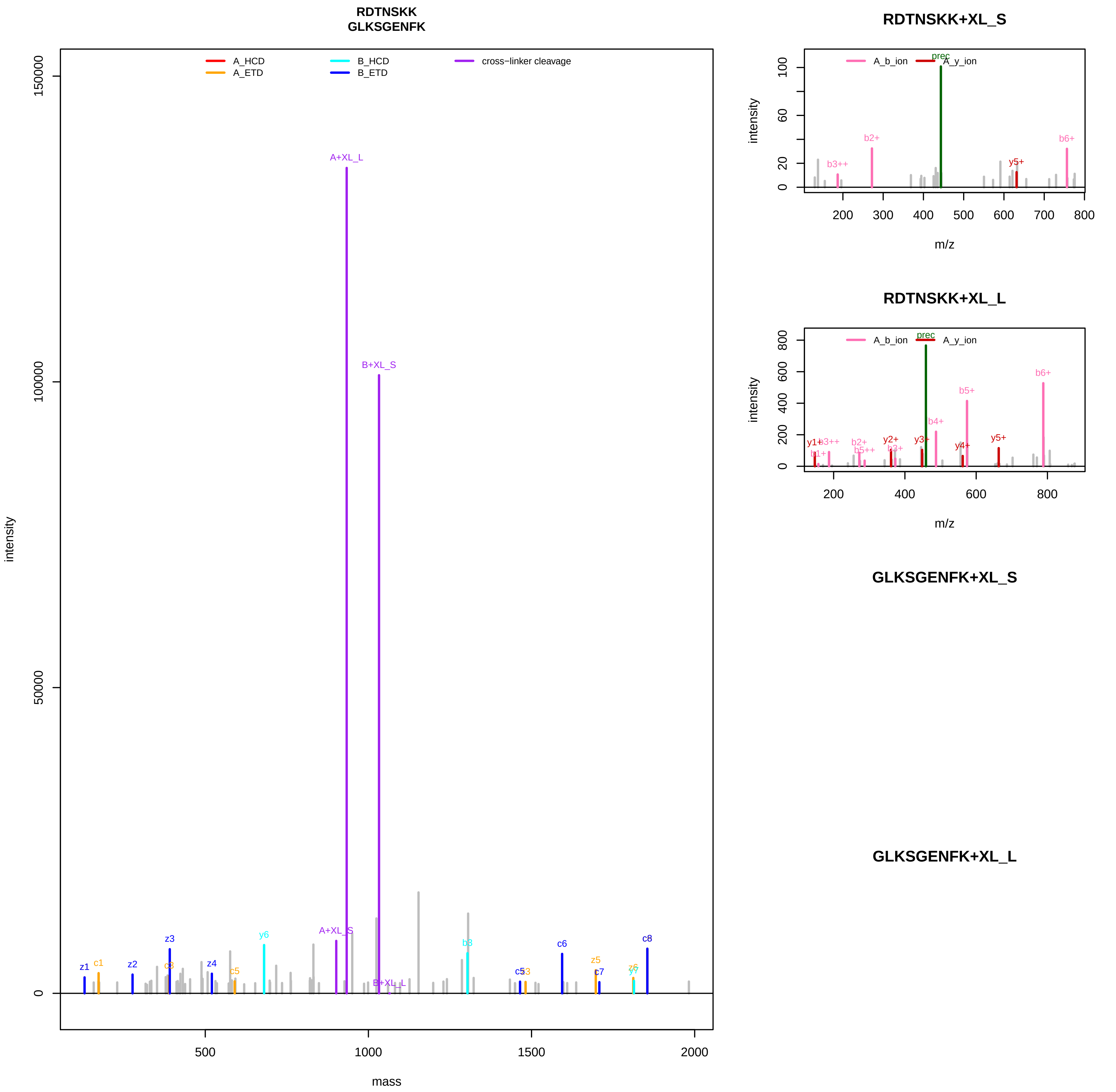

RDTNSKK
GLKSGENFK
A_HCD
A_ETD
B_HCD
B_ETD
cross−linker cleavage
A+XL_L
B+XL_S
A+XL_S
B+XL_L
z1
c1
z2
z3
z4
c5
y6
b3
c5
c6
z5
c7
z6
y7
c8
intensity
mass
0
50000
100000
150000
500
1000
1500
2000
RDTNSKK+XL_S
A_b_ion
A_y_ion
prec
b2+
b3++
y5+
b6+
intensity
m/z
RDTNSKK+XL_L
b1+
y1+
b3++
b2+
b5++
y2+
b3+
y3+
b4+
y4+
b5+
y5+
b6+
GLKSGENFK+XL_S
GLKSGENFK+XL_L
